# Friends and foes: symbiotic and algicidal bacterial influence on *Karenia brevis* blooms

**DOI:** 10.1093/ismeco/ycae164

**Published:** 2024-12-18

**Authors:** Cong Fei, Anne Booker, Sarah Klass, Nayani K Vidyarathna, So Hyun Ahn, Amin R Mohamed, Muhammad Arshad, Patricia M Glibert, Cynthia A Heil, Joaquín Martínez Martínez, Shady A Amin

**Affiliations:** Marine Microbiomics Laboratory, Biology Program, New York University Abu Dhabi, Abu Dhabi 129188, United Arab Emirates; Bigelow Laboratory for Ocean Sciences, East Boothbay, ME 04544, United States; Horn Point Laboratory, University of Maryland Center for Environmental Science, Cambridge, MD 21613, United States; Red Tide Institute, Mote Marine Laboratory and Aquarium, Sarasota, FL 34236, United States; Horn Point Laboratory, University of Maryland Center for Environmental Science, Cambridge, MD 21613, United States; Horn Point Laboratory, University of Maryland Center for Environmental Science, Cambridge, MD 21613, United States; Biology Department, Woods Hole Oceanographic Institution, Woods Hole, MA 02543, United States; Marine Microbiomics Laboratory, Biology Program, New York University Abu Dhabi, Abu Dhabi 129188, United Arab Emirates; Center for Genomics and Systems Biology, New York University Abu Dhabi, Abu Dhabi, 129188, United Arab Emirates; Horn Point Laboratory, University of Maryland Center for Environmental Science, Cambridge, MD 21613, United States; Red Tide Institute, Mote Marine Laboratory and Aquarium, Sarasota, FL 34236, United States; Bigelow Laboratory for Ocean Sciences, East Boothbay, ME 04544, United States; Horn Point Laboratory, University of Maryland Center for Environmental Science, Cambridge, MD 21613, United States; Marine Microbiomics Laboratory, Biology Program, New York University Abu Dhabi, Abu Dhabi 129188, United Arab Emirates; Center for Genomics and Systems Biology, New York University Abu Dhabi, Abu Dhabi, 129188, United Arab Emirates; Mubadala Arabian Center for Climate and Environmental Sciences Center, New York University Abu Dhabi, Abu Dhabi 129188, United Arab Emirates

**Keywords:** dinoflagellates, harmful algal blooms, phytoplankton–bacteria interactions, vitamins, algicidal bacteria, HABs

## Abstract

Harmful Algal Blooms (HABs) of the toxigenic dinoflagellate *Karenia brevis* (KB) are pivotal in structuring the ecosystem of the Gulf of Mexico (GoM), decimating coastal ecology, local economies, and human health. Bacterial communities associated with toxigenic phytoplankton species play an important role in influencing toxin production in the laboratory, supplying essential factors to phytoplankton and even killing blooming species. However, our knowledge of the prevalence of these mechanisms during HAB events is limited, especially for KB blooms. Here, we introduced native microbial communities from the GoM, collected during two phases of a *Karenia* bloom, into KB laboratory cultures. Using bacterial isolation, physiological experiments, and shotgun metagenomic sequencing, we identified both putative enhancers and mitigators of KB blooms. Metagenome-assembled genomes from the *Roseobacter* clade showed strong correlations with KB populations during HABs, akin to symbionts. A bacterial isolate from this group of metagenome-assembled genomes, *Mameliella alba*, alleviated vitamin limitations of KB by providing it with vitamins B_1_, B_7_ and B_12_. Conversely, bacterial isolates belonging to Bacteroidetes and Gammaproteobacteria, *Croceibacter atlanticus*, and *Pseudoalteromonas spongiae*, respectively, exhibited strong algicidal properties against KB. We identified a serine protease homolog in *P. spongiae* that putatively drives the algicidal activity in this isolate. While the algicidal mechanism in *C. atlanticus* is unknown, we demonstrated the efficiency of *C. atlanticus* to mitigate KB growth in blooms from the GoM. Our results highlight the importance of specific bacteria in influencing the dynamics of HABs and suggest strategies for future HAB management.

## Introduction

Harmful algal blooms (HABs) are episodic events in aquatic ecosystems marked by the rapid growth of specific algal species, leading to ecological and economic repercussions that include fish mortality, shellfish poisoning, and disruptions to coastal habitats among other consequences [1–3]. Understanding the factors that drive HABs is essential for creating accurate models, which are crucial for developing predictive capacity for managing blooms [4, 5]. While research has traditionally concentrated on physical and chemical influences like currents, temperature, and nutrients [6–8], the complex interactions between algae and their surrounding microbes, which affect nutrient dynamics and algal growth, remain less understood [9–12]. Therefore, an in-depth examination of the composition and functional interplay within these microbial communities is indispensable to gauge their influence on HAB formation, persistence, and demise.

Microbial communities exert a profound influence on the physiology and dynamics of HABs, including widespread species like *Alexandrium*, *Dinophysis*, *Karenia*, and *Pseudo-nitzschia* [13–16]. These microbes typically inhabit the phycosphere [17], a vital area around phytoplankton cells where diffusion dominates the transport of metabolites released from the cell surface [18]. Therefore, the phycosphere is enriched in algal metabolites that mediate interactions with incoming bacteria, which fall into two broad categories: symbiotic and harmful/algicidal. Beneficial bacteria provide essential nutrients, cofactors, and hormones to phytoplankton. For example, most HA species demonstrate a significant dependence on B vitamins while exhibiting B-vitamin auxotrophy [19, 20]. Vitamin B_12_-producing *Halomonas* sp. was shown to support the growth of *Amphidinium operculatum* and *Porphyridium purpureum* in axenic cultures without vitamin B_12_ [21]. Since some bacteria and archaea produce these vitamins, interactions between HA species and their phycosphere microbes may alleviate vitamin requirements.

Conversely, harmful or algicidal bacteria play important roles in controlling HA species by releasing small molecules or proteins to induce algal cell lysis and/or inhibit their growth [22, 23]. For instance, two algicidal bacterial strains belonging to the *Vibrio* and *Pseudoalteromonas* genera produce a lytic protein, P7, targeting *Alexandrium tamarense* cells [24], while *Thalassospira* sp. produces benzoic acid to lyse *Karenia mikimotoi* cells [25]. Although the use of algicidal bacteria to control HABs has been examined [23], a more detailed investigation of the mechanisms is crucial to fully understand their ecological significance and the implications for marine and coastal systems.

The harmful dinoflagellate *Karenia brevis* (KB) consistently occurs in the Gulf of Mexico (GoM), where it forms blooms that produce neurotoxic brevetoxins. These toxins are responsible for widespread marine mortality, posing significant ecological and economic impacts [26–28]. Despite its ecological significance, research on KB and its associated microbial community has been hampered by challenges such as the lack of viable axenic cultures, phagotrophic behavior, auxotrophy, and the lack of a reference genome due to its estimated extremely large genome size [15, 19, 29–31]. These issues have limited studies to phenotypic observations of algicidal bacteria [15, 31, 32], with minimal insights into microbial mechanisms or connections between lab and natural blooms. For over a decade, studies on bacterial interactions with KB have been largely absent [31]. To address this, we employed xenic cultures, using multiple controls and diverse evidence to draw conclusions. In this study, we leverage shotgun metagenomics alongside *in vitro* and *in situ* experiments to characterize the microbial dynamics that influence KB blooms at the species level. We hypothesized that natural bacterial populations associated with KB blooms in the GoM influence the dinoflagellate’s growth and physiology. Specifically, we define symbiotic and algicidal bacterial candidates from KB-associated communities based on both genomic and experimental evidence and investigate the temporal shifts in these communities throughout the lifecycle of KB blooms. Our findings lay the foundation for a more nuanced understanding of the microbial triggers and regulators of HAB initiation and termination.

## Materials and methods

### Examining the effects of Gulf of Mexico microbial communities on *Karenia brevis*

**Figure 1:**
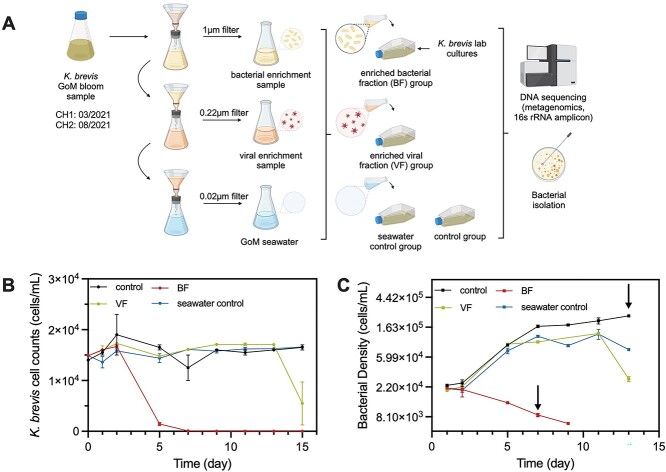
Influence of microbial communities from the GoM on the growth of KB. (A) Experimental schematic illustrating the sequential filtration done to acquire BF and VF microbial communities and seawater (seawater control) fractions from KB GoM blooms with collection times. Subsequently, these fractions were incubated with KB cultures. “Seawater control” and an additional KB culture control (control), which received no additions, served as negative controls. Incubations were ultimately used for DNA sequencing and bacterial isolation. Illustration created with BioRender.com. (B) Cell densities of KB in CH1 for each condition. (C) Total bacterial cell density in CH1 across each condition. Arrows indicate the time points selected for DNA extraction. All error bars represent ranges of duplicate cultures.

To examine how environmental microbial communities from KB blooms affect xenic KB cultures, we amended KB with size-fractionated seawater communities. Briefly, we prefiltered the seawater samples through a 1-μm filter to minimize introducing eukaryotic phytoplankton and non-bacterial heterotrophs. Two toxic KB strains CCMP2229 (Challenge Experiment 1, CH1) and CCMP2228 (Challenge Experiment 2, CH2) were exposed to four conditions: bacterial-viral fractions (<1 μm, BF), viral fraction (<0.2 μm, VF), particle-free seawater (<0.02 μm, seawater control), and a non-inoculated xenic KB control (control). The xenic KB was at late stationary phase, as determined by its growth rate, reflecting late bloom stages (Fig. 1A). Full details of strains, growth conditions, and size fraction are provided in Supplementary Methods. The metadata for seawater samples collected for CH1 and CH2 are listed in Table S1.

### Monthly environmental sampling

Surface seawater samples (2–5 l) were obtained monthly from Station EH25 (latitude 26.7106, longitude −82.3345) from December 2020 to August 2021. These were prefiltered (100, 10, and 3 μm) to remove particulates, followed by filtration through 0.22 μm membranes to capture free-living bacterial communities. Filters were preserved in 2 ml cryovials with liquid nitrogen and stored at −80°C for subsequent analysis. A 5 ml aliquot was treated with Lugol’s iodine solution for algal identification and counted via a Sedgewick-Rafter counting chamber under a Zeiss Primo-Start Compound Microscope. Sample metadata is provided in Table S2.

### 16S ribosomal ribonucleic acid amplicon and shotgun metagenomic data analysis

Deoxyribonucleic acid (DNA) extraction, quantification, and sequencing, including 16S ribosomal ribonucleic acid (rRNA) amplicon and shotgun metagenomic approaches, were performed as described in Supplementary Methods and Table S3. The microbial community composition derived from 16S rRNA amplicon sequencing was analyzed using the Quantitative Insights into Microbial Ecology (QIIME2) pipeline [33] (Supplementary Methods), with ASVs read counts provided in Table S4. Digital normalization and sequencing quality control were conducted using Khmer [34] and the Read_QC module in metaWRAP [35] for metagenomic sequences. Taxonomic identification was performed with Kraken 2 [36], and results were visualized in Pavian [37]. Community composition was depicted using bar plot and principal coordinates analysis (PCoA) plots in R. Quality control and trimming procedures are detailed in Supplementary Methods. Co-assembly and binning were performed for the CH1 and CH2 experiments using the metaWRAP pipeline with the MEGAHIT assembler (v1.1.2) and default settings [38]. Further details are provided in the Supplementary Methods.

### Annotation, quantification, and function enrichment of metagenome-assembled genomes

Taxonomic assignment for each MAG was performed using GTDB-Tk v0.2.2 against the Genome Taxonomy Database (GTDB) release 202 [39]. To determine the Average Nucleotide Identity (ANI) between metagenome-assembled genomes (MAGs) from CH1 and CH2, we employed a BLAST-based approach (ANIb) using pyani v0.2.10 (https://github.com/widdowquinn/pyani) [40], with a threshold of 96% identity for significance. Quantification of the MAGs was achieved using two distinct methodologies: Salmon and BBsplit [41, 42] (Supplementary Methods). For functional enrichment analysis, seventeen MAGs and three genomes of isolated bacteria were analyzed using the anvi’o v7 pangenomic workflow [43], as detailed in Supplementary Methods.

### Bacterial isolation, identification, whole genome sequence, and secondary metabolites prediction

Bacterial strains were isolated from CH1 samples, and genomic DNA was extracted using the EZNA Tissue DNA Kit. Full-length 16S rRNA genes were amplified for maximum likelihood phylogenetic analysis. Detailed methods are provided in Supplementary Methods. Whole-genome sequencing of three bacterial strains was performed using Illumina NovaSeq 6000 for short reads and PacBio Sequel II for long reads, employing the DNA CLR library at Novogene (HK, China). Quality control and hybrid assembly were done with Unicycler (v0.4.0; Supplementary Methods). Biosynthetic Gene Clusters (BGCs) were predicted using the AntiSmash 6.0 bacterial version with default settings and all additional features activated [44]. A “strict” filter was applied to select well-characterized clusters. These BGCs were then compared against the MIBiG database for accurate annotation verification and detailing.

### Assessing bacterial influence on *Karenia brevis*: *in vitro* and *in situ* assays

Two sets of *in vitro* co-culture experiments were conducted between bacterial isolates and xenic KB CCMP2229 cells. Initially, xenic KB cells in the early to mid-exponential phase were inoculated into fresh L1-Si media at 1000 cells/ml. Overnight cultures of *C. atlanticus* CE21 and *P. spongiae* CE15 (26°C, 180 rpm) were inoculated into the culture at a concentration of 1x10^6^ cells/ml. The protease experiment was conducted on *P. spongiae* CE15, modified from previously described methods [45], with details provided in Supplementary Methods. In a parallel experiment, KB cells pre-incubated in vitamin-depleted (thiamine, biotin, and cyanocobalamin) L1-Si media for 20 days were transferred to fresh, vitamin-depleted media at the same density of 1000 cells/ml, with *M. alba* CE5 added at 1x10^6^ cells/ml. For controls, the same KB cultures were supplemented with these vitamins, either individually or in combination, at the same concentrations as in the L1-Si medium. All experiments were performed in triplicates. The growth of KB was monitored by a Turner 10-AU fluorometer, calibrated for in vivo chlorophyll a fluorescence, and recorded as relative fluorescence units (RFU). Algicidal ratios were calculated using the formula:


$$ \mathrm{Algicidal}\ \mathrm{ratio}\ \left(\%\right)=\left({\mathrm{RFU}}_{\mathrm{co}\mathrm{ntrol}}-{\mathrm{RFU}}_{\mathrm{co}-\mathrm{culture}}\right)/{\mathrm{RFU}}_{\mathrm{co}\mathrm{ntrol}}\ \mathrm{x}\ 100 $$

*In situ* experiments were conducted during the ECOHAB process cruise to assess the effects of *C. atlanticus* on KB growth. Seawater samples were collected and inoculated with *C. atlanticus* at 1 × 10^6^ cells/ml in triplicate flasks. KB cell counts were recorded at 0, 16, and 24 hours under a Zeiss Primo-Start Compound Microscope. Full details of sample collection and incubation conditions are provided in Supplementary Methods.

### Global distribution of *espI*

The amino acid sequence of *espI* from *Pseudoalteromonas* sp. A28, known for its algicidal extracellular serine protease activity as detailed by Kohno et al. (2007) [61], was analyzed for similarity against the Tara Oceans Microbiome Reference Gene Catalog version 1. This analysis was conducted via the Ocean Gene Atlas portal (https://tara-oceans.mio.osupytheas.fr/ocean-gene-atlas/) [46]. A stringent e-value threshold of 1e^−50^ guided homolog identification to ensure high specificity. Geographical distribution and taxonomic abundance of homologs in surface water samples across the 0.22–3 μm size fractions were visualized as donut plots on a world map, using the “maps” and “ggplot2” packages in R [47, 48].

## Results

### Gulf of Mexico microbial communities contributed to the mortality of *Karenia brevis*

We hypothesized that natural bacterial populations associated with the KB blooms in the GoM influence the dinoflagellate’s growth and physiology and may lead to mortality. To test this, we size-fractionated KB bloom water from the GoM through multiple filters to obtain distinct fractions as described in the methods (Fig. 1A, Table S1). Each fraction was then used to “challenge” KB CCMP2229 cultures grown to late stationary phase to resemble a late bloom stage; this experiment was termed challenge experiment 1 (CH1). While KB cell densities in the seawater control and non-inoculated culture control showed no significant change over two weeks, cell densities in cultures amended with the bacterial (and viral) fraction group (BF) declined rapidly within 2–5 days of inoculation from an initial 1.49 x 10^4^ cells/ml to 1.44 x 10^3^ cells/ml on day 5 and to no observable KB cells from day 7, indicating the presence of an algicidal agent (Fig. 1B). Similar results were observed in cultures inoculated with the viral fraction group (VF) between days 13 and 15 after inoculation, where KB cell density declined to 5.49 x 10^3^ cells/ml, indicating the presence of an algicidal agent that was significantly delayed relative to BF. A decrease in bacterial cell densities aligning with the decline in KB numbers was also observed in the BF and VF treatments (Fig. 1C). This may result from limited carbon availability in the late stationary phase of KB, compounded by the release of brevetoxins during cell lysis, which can suppress bacterial populations [49, 50].

To ensure the reproducibility of the CH1 experiment, we conducted a repeat experiment (termed challenge experiment 2, CH2) approximately four months later, using bloom water from the same site as CH1, and a different strain of KB CCMP2228 (Table S1). Remarkably, the additions of microbial communities in BF and VF resulted in consistent declines of KB and bacterial cell abundances in both CH1 and CH2 (Fig. S1), suggesting the algicidal agents are ubiquitous in bloom waters in the GoM independent of time and KB strains.

### Microbial community composition across treatments

**Figure 2:**
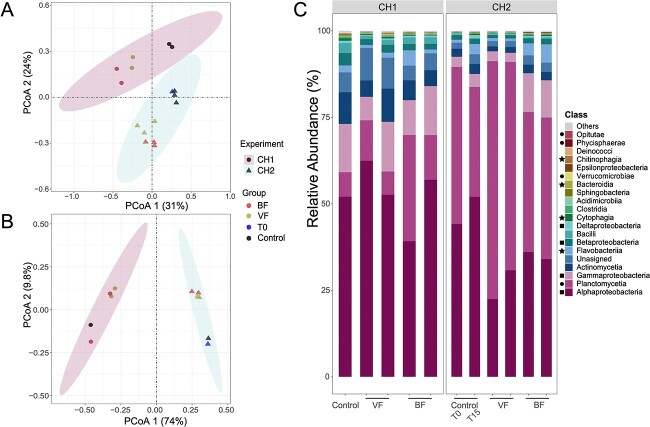
Bacterial diversity and composition in CH1 and CH2 experiments. PCoA based on 16S rRNA ASVs (A) and shotgun metagenomes (B) for CH1 (circles) and CH2 (triangles) experiments. Dots are colored according to their respective groups, with 95% confidence ellipses provided for each experiment (*P* = .001 for A, *P* = .002 for B). (C) Bar plot depicting the taxonomic composition of the top 20 bacterial classes in CH1 and CH2 based on metagenomic data. Key bacterial groups are highlighted: Pseudomonadota (squares), PVC group (circles), and FCB group (stars).

We employed 16S rRNA amplicon sequencing and metagenomics on DNA samples extracted from BF, VF, and control groups for both CH1 and CH2, and an additional sample from CH2 representing the original KB culture microbiome immediately collected prior to inoculation with GoM fractions (ControlT0). This enabled us to characterize the taxonomic composition and abundance changes in bacterial communities in each treatment at the start and end points of the experiments. PCoA analysis of 16S rRNA amplified sequence variants (ASVs) and shotgun metagenomes showed distinct clustering between CH1 and CH2 with a statistically significant confidence level at 95% (*P* < .01), indicating significant differences in the microbial community composition added to KB in CH1 and CH2 and between the KB strains (Fig. 2A and B). Interestingly, the microbial diversity of ControlT0 clustered with Control after 15 days of incubation in CH2, indicating a stable native microbial community in KB cultures over 15 days (Fig. 2A and B). BF and VF groups clustered separately from controls (*P* = .002), indicating a significant shift in the microbial community upon the addition of GoM bloom waters to the cultures (Fig. S2A). Despite some taxonomic differences between 16S rRNA and metagenomic analyses arising from the use of different databases for each (Silva 138 and Kraken 2, respectively), as well as compositional biases inherent in 16S rRNA gene amplification due to PCR [51], beta diversity consistently demonstrated significant shifts in microbial communities patterns for BF and VF versus controls across both datasets. Notably, Gammaproteobacteria increased in CH1 and CH2 combined average abundance in BF relative to controls (16S rRNA_control_: 8.4%, 16S rRNA_BF_: 15.3%; metagenome_control_: 6.9%, metagenome_BF_: 11.5%), while Alphaproteobacteria were more abundant in controls (16S rRNA_control_: 65.8%, 16S rRNA_BF_: 53.6%; metagenome_control_: 49.4%, metagenome_BF_: 41.6%; Figs 2C and S2B).

### Identification of metagenome-assembled genomes of potential *Karenia brevis* symbiotic and algicidal bacteria

**Figure 3:**
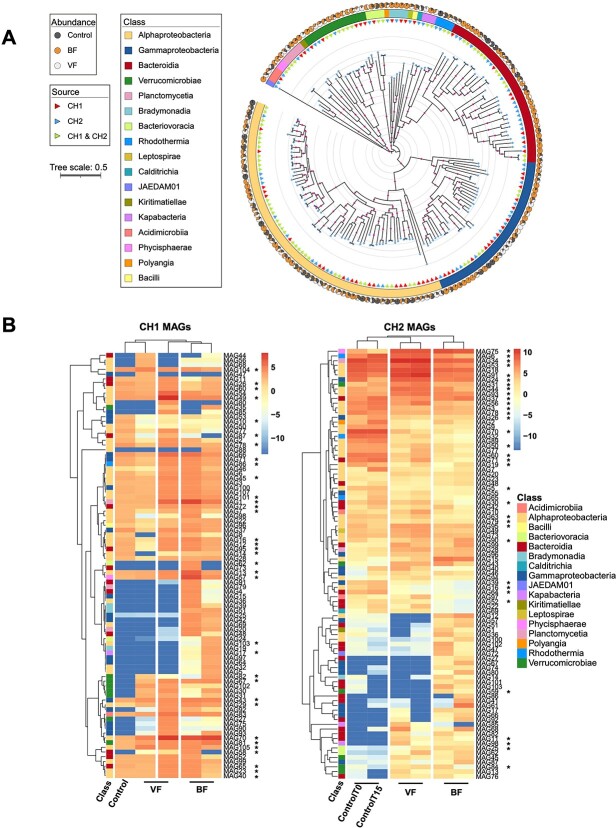
Phylogenomic relationships and relative abundance of bacterial MAGs across CH1 and CH2. (A) Unrooted maximum-likelihood phylogenomic tree derived from 71 conserved bacterial markers in anvi’o v7, visualized with iTOL v5. Outer track: pie charts represent MAG abundance across the control, BF and VF groups. Middle track: colors indicate bacterial class. Innermost track: triangles denote MAG source (CH1 or CH2). Circle dots at branches indicate bootstrap values >95%, and diamond dots at nodes represent individual MAGs. (B) MAG relative abundances in CH1 (left) and CH2 (right) experiments. Abundances are represented as z-score transformed positive or negative values. The class of each MAG is represented by the left column in each heatmap. ^*^ indicates consensus MAGs between CH1 and CH2.

To investigate the functional potential of bacteria in CH1 and CH2, we assembled 90 MAGs from CH1 and 91 MAGs from CH2 (>80% completeness, <10% contamination) that represented 82.7% of total metagenomic reads (Table S5). A phylogenetic tree encompassing all 181 MAGs revealed their taxonomic affiliation with 17 distinct classes (Fig. 3A), with Alphaproteobacteria (37.6%), Bacteroidia (19.3%), Gammaproteobacteria (18.8%), Verrucomicrobia (7.7%), and Planctomycetia (2.8%) being the most prevalent. Furthermore, a subset of 34 consensus MAGs shared across both CH1 and CH2, were considered nearly identical (average nucleotide identity [ANI] >96%) [52] (Fig. 3A and S3, Tables S6), with Alphaproteobacteria constituting 50% of consensus MAGs. The consistent presence of these MAGs indicates they are either common to the KB microbiome or resilient bacteria in the GoM that survive during KB blooms.

To assess the potential contribution of MAGs to KB growth dynamics in CH1 and CH2, their relative abundance in CH1 and CH2 was examined using metagenomic read recruitment (Fig. 3B). We hypothesized that a group of 28 consensus MAGs commonly present in all CH controls may be core to the KB microbiome and potentially symbiotic (control MAGs, Table S6). Notably, eight belong to the *Roseobacter* clade within the Rhodobacteraceae family, known for being algal symbionts [15, 48, 49]. We also hypothesized that a group of 50 MAGs exclusive to the BF and/or VF groups and absent in controls, originated from GoM seawater (non-control MAGs). Some of these MAGs may be algicidal, as their addition caused KB mortality. These MAGs mainly belonged to the phylum Bacteroidota (28%) and the class Gammaproteobacteria (26%; Table S7). For control MAGs, functional enrichment of the 28 MAGs showed the prevalence of genes involved in NADH dehydrogenase (ubiquinone), Fe-S protein/flavoprotein complex, tryptophan biosynthesis, and cobalamin biosynthesis (unadjusted *P* < .005, adjusted q < 0.05; Table S8). Among cobalamin biosynthesis genes, nine enriched genes are involved in aerobic cobalamin synthesis (*cobB*, *cobF*, *cobH-N*), suggesting a potential to provide vitamin B_12_ to KB, consistent with the fact that KB is auxotrophic for B vitamins [18, 19].

### Positive correlation between *Karenia brevis* density and its potential symbiotic metagenome-assembled genomes during Gulf of Mexico blooms

**Figure 4:**
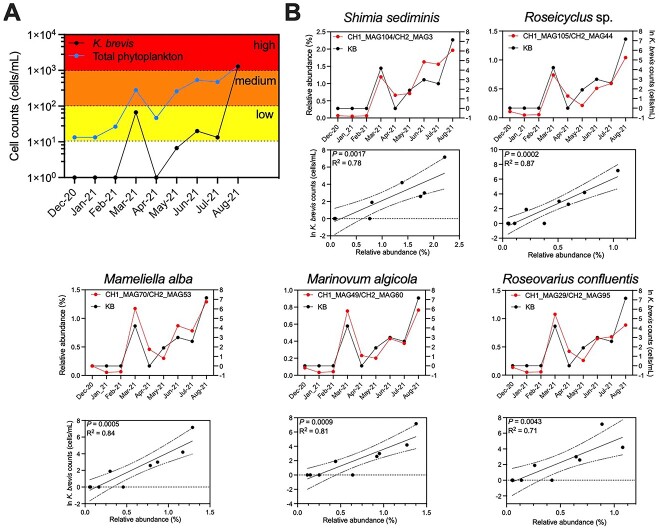
Abundance of potential KB symbiotic MAGs during a GoM KB bloom. (A) Monthly cell counts of KB and total phytoplankton collected at station EH25 in the GoM. Background shade colors represent different bloom intensity levels as defined by the Florida Fish and Wildlife Research Institute (https://myfwc.com/research/redtide/statewide/). (B) Top graphs: Relative abundance of 5 potential symbiotic MAGs based on metagenomic read recruitment overlaid with KB cell counts. Bottom graphs: Linear regression fit of MAG relative abundance and KB cell counts. Each dot represents a monthly sample. The solid lines represent the linear fit, while the dotted lines depict the 95% confidence intervals of the fit.

We hypothesized that if some of the control MAGs were KB symbionts that potentially provided it with essential cofactors like vitamins, their abundance trends in nature should closely follow KB. To examine this possibility, we collected monthly DNA samples near Boca Grande in the GoM from December 2020–August 2021, coinciding with a KB bloom starting in March 2021 (Table S2). KB was not detected from December 2020 to February 2021, followed by a low-level bloom (10–100 cells/ml) in March and a high-bloom event from May 2021 onwards, where KB cell densities reached >1000 cells/ml (Fig. 4A). These samples were used to acquire monthly shotgun metagenomic samples.

The relative abundance of control MAGs was compared against KB cell densities across the monthly samples. While most MAGs had no significant correlation with KB, five MAGs displayed a strong positive correlation with KB throughout the time series (R^2^ ranging from 0.71 to 0.87, *P* < .02; Table S9, Fig. 4B). All five MAGs belong to the *Roseobacter* clade (Table S5), members of which are symbionts of many phytoplankton lineages [16, 53, 54]. More importantly, these MAGs constituted a significant fraction of bacterial reads during the bloom peak in August 2021, with an average relative abundance of 1.19% of all metagenomic reads in the 0.22 μm size fraction (see Materials and methods), compared to 0.07% for the 50 MAGs absent in controls (*P* = .0002; Table S10). For example, *Mameliella alba* (CH1_MAG70) reached a peak relative abundance of 1.3%; this is a significant presence, representing a single genome/species, suggesting a bacterial bloom concurrent with the KB bloom. While 21.4% (6 out of 28) of control MAGs correlated with KB (R^2^ > 0.7), only 6% (3 out of 50) of MAGs absent in control cultures, potentially representing algicidal or opportunistic bacteria, showed a positive correlation to KB abundance (Table S9). This lack of correlation during the initial phase, combined with their low abundance at the bloom peak, likely reflects the tendency of algicidal bacteria to become more abundant and active in the later stages of the bloom [22].

### Recovering algicidal and symbiotic bacterial isolates representing metagenome-assembled genomes

**Figure 5:**
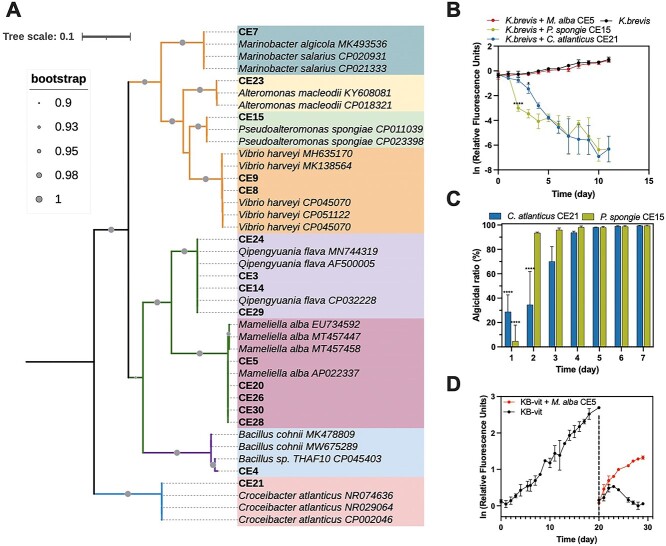
Interaction of KB with select bacterial strains from CH1. (A) Maximum-likelihood phylogenetic tree based on partial 16S rRNA sequences, depicting the taxonomy of bacteria isolated from CH1. Bacterial classes are differentiated by branch colors, while different genera are highlighted in different background colors. Bacterial isolates are in bold. Circle dots at branches indicate bootstrap values >90%. (B) Co-culture experiments of KB with three bacterial isolates. *Karenia* growth was monitored using relative chlorophyll a fluorescence (RFU). Significant differences of RFU are labeled where they first occur, with *P* < .05 (^*^), and *P* < .0001 (^*^^*^^*^^*^). (C) Algicidal ratios exhibited by *P. spongiae* CE15 and *C. atlanticus* CE21 in co-culture with KB. ^*^^*^^*^^*^P < .0001. (D) Co-culture of KB under vitamins B_1_, B_7_ and B_12_ limitation in the presence or absence of *M. alba* CE5. KB was cultured under vitamin-limited conditions for 20 days (left curve) followed by fresh culture inoculation into vitamin-limited media with or without *M. alba* CE5 (right curves). All error bars represent standard deviations from triplicate experiments.

To study the potential role of the MAGs highly correlated with KB as well as potentially algicidal MAGs, bacteria were isolated from CH1 (Fig. 1A). Sixteen strains were cultured and identified across eight genera, spanning four distinct classes: Gammaproteobacteria, Alphaproteobacteria, Bacilli and Flavobacteriia (Fig. 5A). Complete genomes of select bacterial isolates were sequenced if the isolate’s 16S rRNA based taxonomy matched control/non-control MAGs taxonomy**.***M. alba* CE5 genome and CH1_MAG70 from control cultures had an ANI value of 99.94%, indicating they are likely the same species. *Croceibacter atlanticus* CE21 genome and CH1_MAG91 had an ANI of 99.98%, while *Pseudoalteromonas spongiae* CE15 genome and CH1_MAG47 had an ANI of 78.53%, with both MAGs absent in controls.

To examine the effect of these bacteria on KB, we co-cultured each strain with KB. *P. spongiae* CE15 and *C. atlanticus* CE21 inhibited the growth of KB CCMP2229 within 48 hours (Fig. 5B), with algicidal ratios surpassing 80% from day 4 onwards, underscoring their potent algicidal activity against KB (Fig. 5C). Conversely, *M. alba* CE5 did not demonstrate any discernible impact on the growth of KB when grown in nutrient- and vitamin-rich media (Fig. 5B). Since genes in the cobalamin biosynthesis pathway were enriched in control MAGs, including *M. alba* CH1_MAG70 (Table S8), and KB is an auxotroph for vitamins B_1_, B_7,_ and B_12_ [19], which are putatively synthesized by *M. alba* CE5 based on genomic evidence (Table S11), we hypothesized that *M. alba* may supply these vitamins to KB when they are co-limiting. To test this hypothesis, we cultured KB without these vitamins for 20 days, creating a vitamin-deplete environment. Subsequently, *M. alba* CE5 was inoculated under vitamin-depleted conditions. While the density of vitamin-deplete KB cultures declined after 3 days, cultures without vitamins that were amended with *M. alba* CE5 exhibited similar growth rates to vitamin-replete KB (*μ_k. brevis + vitamins_* = 0.14 ± 0.004; *μ*_co-culture_ = 0.15 ± 0.012; Fig. 5D). Additionally, we supplemented the vitamin-deplete KB cultures with each of the three vitamins individually and in combination. The growth rate of KB matched that of cultures supplemented with *M. alba* only when all three vitamins were added (Fig. S4), indicating that *M. alba* CE5 synthesizes these vitamins for KB growth.

### Potential mechanisms of action of symbiotic and algicidal bacteria affecting *Karenia brevis* growth

**Figure 6:**
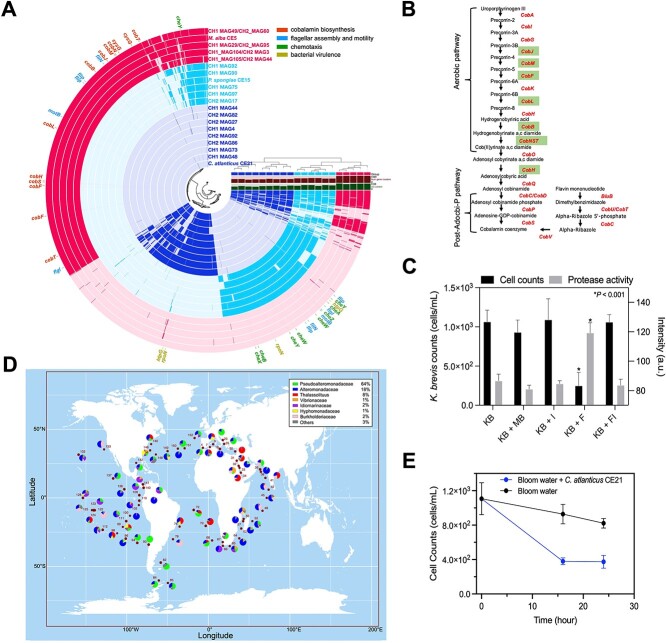
Deciphering the molecular mechanisms of putative symbiotic and algicidal bacteria associated with KB*.* (A) Pangenomic analysis of 2957 gene clusters across 20 MAGs and isolate genomes. The central dendrogram shows hierarchical clustering based on gene cluster presence (dark) or absence (opaque). Each track represents a MAG or genome, while track colors indicate the putative type of interaction with KB (pink = symbiotic, light blue and dark blue = algicidal). Important genes from different genomic groups are annotated around the pangenome’s periphery and colored into four distinct groups based on their functions. Only gene clusters present in ≥5 MAGs/genomes are shown; unique gene clusters are not displayed. (B) Cobalamin biosynthesis pathway in putative symbiotic MAGs and *M. alba* CE5. Gene names in red are fully present in the pink tracks in (A), while names highlighted in green are absent in putative algicidal genomes. (C) Inhibition of KB by *P. spongiae* CE15 extracellular protease. KB cell counts and protease activity were measured under conditions of filtrate (F), protease inhibitor PMFS (I), and marine broth (MB). *P* values were evaluated through one-way ANOVA, followed by Dunnett’s multiple-comparison test. (D) Geographic distribution of the algicidal serine protease, EspI, from *P. spongiae* CE15 in the Tara Oceans database. Colors in the donut plots represent the taxonomic distribution of this protein in different taxa in surface waters (from 0.22 to 3 μm). Numbers denote Tara Oceans stations. (E) Inhibition of KB natural populations during a bloom in the GoM by *C. atlanticus* CE21. Water was collected at station TC16 and incubated for 24 hours (see Materials and methods). Error bars represent the SD of triplicate incubations.

To elucidate the functional mechanisms that enable *C. atlanticus* and *P. spongiae* to kill KB and *M. alba* to boost KB growth, we constructed a pangenome of their genomes along with related MAGs, categorized by their interaction with KB: (i) *M. alba* CE5 genome and four MAGs from control MAGs that positively correlated with KB blooms as potential symbionts (Fig. 4); (ii) *P. spongiae* CE15 genome and five MAGs from the same order, Enterobacteriales, absent in CH controls and hypothesized to be algicidal; (iii) *C. atlanticus* CE21 genome and eight closely-related Flavobacteriales MAGs, missing in CH controls and hypothesized to be algicidal (Fig. 6A). Functional enrichment analysis included gene clusters present in at least five MAGs/genomes, reflecting the minimum group size of the individual groups. In the two putative algicidal groups (b and c), only the RNA polymerase sigma-54 factor (*rpoN*) and the heat-shock protein gene *htpG* were consistently present and linked to bacterial virulence [55, 56], while these genes were absent in group a (Fig. 6A, Table S12). The *P. spongiae* group was enriched in chemotaxis-related genes (*cheA*, *cheB*, *cheW-Z*), suggesting a specific chemotactic response used to track algal cells. On the other hand, the absence of genes associated with flagellar assembly and motility (*fliN*, *fliP*, *flgI*, and *motB*) in the *C. atlanticus* group suggests that these bacteria are non-motile [57]. Interestingly, the *M. alba* group possessed the complete pathway for Cobalamin/vitamin B_12_ biosynthesis, while both algicidal groups only possessed an incomplete pathway, indicating only the putative symbiotic genomes are capable of synthesizing cobalamin (Fig. 6A and B). These findings support our finding that *M. alba* supports KB growth in vitamin-limited conditions (Fig. 5D), hinting at a similar role for the other four *Roseobacter* MAGs strongly correlating with KB in bloom waters (Fig. 4B).

Since the *Pseudoalteromonas* genus contains many algicidal bacteria [22, 58–60], we mined the genome of *P. spongiae* CE15 for potential algicidal genes based on existing knowledge. A serine protease gene in *P. spongiae* CE15 was identified with 70.4% amino acid identity to *espI*, a gene associated with the algicidal activity of *Pseudoalteromonas* sp. strain A28 against the diatom *Skeletonema costatum* [60, 61], while not being common within the pangenome for two putative algicidal groups (Fig. 6A). This similarity suggests that the serine protease from *P. spongiae* might contribute to its ability to kill KB (Fig. 5B and C). To test this hypothesis, we applied a cell-free filtrate with active extracellular proteases from *P. spongiae* CE15 to target KB. Inhibition of protease activity with phenylmethanesulfonyl fluoride (PMFS) revealed that only the active filtrates had significant protease activity compared to controls (*P* < .001; Fig. 6C). Additionally, KB cell density significantly decreased by adding the active filtrates, showing a marked reduction 5 hours post-inoculation (0.25 x 10^3^ cells/ml) compared with the control (1.07 × 10^3^ cells/ml, *P* < .001; Fig. 6C), indicating that the serine protease may play a key role in the antagonistic effect of *P. spongiae* CE15 against KB. Mapping the abundance of *espI* across the Tara Oceans database shows it is widespread but rare relative abundance ranging from 6.39 × 10^−9^ to 2.61 × 10^−5^, expressed as the ratio of *espI* reads to total reads. Taxa that harbor this gene appear restricted to mostly the Pseudoalteromonadaceae (64%) and Alteromonadaceae (18%) families (Fig. 6D).

While *C. atlanticus* extracellular substrates have been shown to inhibit marine diatom growth [62], the specific active components remain unidentified. Our genome analysis of *C. atlanticus* CE21 revealed potential gene clusters related to algicidal functions, including protease and quorum sensing genes [57, 63]. Additionally, the genome contains a partial Type III PKS cluster, sharing 61% similarity with the *phID* gene involved in the biosynthesis of the algicidal molecule 2,4-diacetylphloroglucinol (DAPG) [64], though other genes in the DAPG biosynthesis pathway are missing. While direct evidence of *C. atlanticus*’ algicidal mechanism is lacking, our studies confirm its ability to suppress KB growth in both cultures (Fig. 5C) and natural blooms. In onboard incubations, adding *C. atlanticus* CE21 to bloom water efficiently reduced KB cell numbers within 16 hours, from 1.11 x 10^3^ cells/ml to 3.79 x 10^2^ cells/ml, while bloom water lacking *C. atlanticus* CE21 exhibited negligible changes in cell density (Fig. 6E). These findings further underscore the potential role of *C. atlanticus* in mitigating *Karenia* blooms.

## Discussion

In aquatic ecosystems, some bacteria within the phycosphere engage in mutualistic exchanges with phytoplankton, facilitating nutrient acquisition and growth. In laboratory studies, members of the *Roseobacter* group have demonstrated beneficial interactions with various phytoplankton lineages, including dinoflagellates [53, 54, 65], by sharing essential metabolites such as trace metals, vitamins, and hormones [16, 21, 66]. The observation that several *Roseobacter* group MAGs strongly correlate with KB during blooms in the GoM helps narrow our search for symbiotic bacteria from the entire microbiome to this specific MAGs group.

While scarce information exists on the interactions between these specific taxa and phytoplankton, we have shown that one member of the above group, *M. alba,* benefits KB during vitamins colimitation. Previous studies showed this species was consistently isolated from diatoms and dinoflagellates [67–69] and led to a significant algal growth rate enhancement of multiple dinoflagellates, including the HA species *Alexandrium catenella* [67]. However, the mechanisms of this growth enhancement are unknown. Our work provides evidence that *M. alba* alleviates vitamin limitation of KB by providing three vitamins (Figs 5D, 6B, and S4, Table S11). To our knowledge, this is the first evidence of KB reliance on a putative bacterial symbiont to acquire vitamins, for which it is auxotrophic [19–21]. The high relative abundance of *M. alba* reads, comprising ~1.3% of the total microbial community at bloom peak, underscores its putative critical role for *Karenia* blooms. No studies have examined vitamin bioavailability in the GoM and future work needs to examine the role vitamins play in HAB events in the GoM and beyond [70]. Overall, these findings warrant a reevaluation of microbial roles in HAB ecology, suggesting that these symbiotic interactions may be crucial in modulating bloom dynamics.

While little is known about the role of beneficial bacteria in sustaining algal hosts, significantly more is known about algicidal bacteria. Previous studies have shown that introducing algicidal strains can cause substantial mortality in various HAB species, including KB, along with significant shifts in the microbial community [14, 15, 71]. Our work demonstrates that introducing bloom microbial communities into KB cultures at the late stationary phase can induce mortality in KB and that specific members of those communities are able to induce mortality in KB in the laboratory and in the field. The mechanisms underpinning algicidal activity vary wildly. For example, *Formosa* spp. S03 requires direct contact with KB to induce mortality, whereas *Cytophaga* sp. 41-DBG2 releases heat-labile algicides [31]. Other bacteria, such as *Kordia algicida*, release extracellular proteases to induce mortality in diatoms [45, 72]. *C. atlanticus* has been shown to impair diatom growth through two distinct mechanisms: cell cycle/division inhibition in a centric diatom direct cell–cell attachment and cell lysis in toxigenic diatoms [62, 73]. For KB, *C. atlanticus* CE21 might use similar tactics to antagonize algal cells. Genomic analysis reveals *C. atlanticus* CE21 lacks genes for motility and chemotaxis, hinting at the reliance on colonization and attachment in its interactions with KB. Furthermore, the potential for algicidal-molecule production, possibly involving extracellular proteases, quorum sensing signals, and/or lipid-based algicides, may exist within certain gene clusters that have not been confirmed or characterized. Future work should focus on elucidating the algicidal mechanisms in *C. atlanticus*, which can inform the breadth of its algal-killing efficacy and enable HAB mitigation. This is particularly important as *C. atlanticus* is able to infect a wide range of phytoplankton species, including diatoms and dinoflagellates, and appears to be mostly actively killing toxigenic species [62, 73].

Gammaproteobacteria, especially the genus *Pseudoalteromonas*, have been shown to negatively correlate with several HAB species, e.g. *Noctiluca scintillans* and *A. tamarense* [58, 59, 74]. Our study highlights the strong algicidal properties of *P. spongiae* CE15 against KB, further supported by the identification of a gene homologous to the globally distributed algicidal gene *espI* in *Pseudoalteromonas*. This gene, identified in *Pseudoalteromonas* sp. strain A28, encodes an extracellular serine protease EspI [60, 61], which shows algicidal activity against *Skeletonema costatum* when expressed in *Escherichia coli*. Our findings confirm that extracellular proteases from *P. spongiae* CE15 are capable of killing KB, suggesting that similar mechanisms may be at play. Despite the identification of numerous *Pseudoalteromonas* strains harboring EspI or homologous enzymes, the comprehensive impact and application of these proteases on bloom dynamics remains to be elucidated [75, 76]. Bacteria such as *C. atlanticus* and *P. spongiae* could be key in controlling HABs. Future studies should delve into the mechanisms that enable these bacteria to gain a foothold in the phycosphere. Finally, understanding how these bacteria interact with beneficial counterparts and the role of viral-phages in algal bloom dynamics is crucial for comprehending bloom initiation and dissipation.

Given the challenges of maintaining KB in axenic conditions due to its complex auxotrophy and phagotrophy [19, 29, 30], research on its bacterial interactions has never been studied under axenic conditions for bacterial interactions and stalled since 2008 [15, 31, 77], despite its ecological importance. In this context, our study highlights the value of xenic cultures as a viable alternative. We employed an approach for investigating bacterial interactions in xenic cultures by using multiple controls and integrating various lines of evidence to support our conclusions. Importantly, we show that xenic cultures can effectively mimic real-world conditions, consistently replicating the algicidal effects of *C. atlanticus* on KB observed in both laboratory cultures and natural blooms in the GoM, underscoring the benefits of xenic cultures for studying these interactions. Through this approach, we provide novel insights into the intricate relationships between KB and its associated microbial community, a topic of significant ecological interest. We show that KB maintains a dominant microbiome that is crucial for its survival and can provide it with required cofactors, such as vitamins. Our work identifies specific algicidal bacteria within the *Croceibacter* and *Pseudoalteromonas* genera that induce KB mortality and thereby potentially regulate HAB dynamics. These findings not only contribute to our understanding of the complex ecological interactions within HABs but also open new avenues for innovative management strategies, emphasizing the importance of microbial dynamics in marine ecosystems. The revelation of these dual roles of microbial communities in supporting and controlling KB underscores a sophisticated balance of interactions, presenting a significant step forward in our understanding of HAB lifecycle and management.

## Supplementary Material

SUPPLEMENTARY_INFORMATION_ycae164

supplementary_Table_ycae164

## Data Availability

16S rRNA amplicon reads of Challenge experiments are deposited in NCBI under the Bioproject PRJNA1070992. Shotgun Metagenomic reads of the Challenge experiments and monthly field samples are deposited in NCBI under the Bioproject PRJNA1077797. The 16S rRNA genes of isolated bacteria are under the GenBank PP348096-PP348111. The genomes of isolated bacteria are under the Bioproject PRJNA1052587. Metagenomically assembled genomes and their annotations are available on Zenodo (https://zenodo.org/records/10579931).

## References

[1] Grattan LM , Holobaugh S, Morris JG Jr. Harmful algal blooms and public health. *Harmful Algae* 2016;57:2–8. 10.1016/j.hal.2016.05.003 PMC5016795 27616971

[2] Landsberg JH . The effects of harmful algal blooms on aquatic organisms. *Rev Fish Sci* 2002;10:113–390. 10.1080/20026491051695

[3] Anderson DM , Glibert PM, Burkholder JM. Harmful algal blooms and eutrophication: nutrient sources, composition, and consequences. *Estuaries* 2002;25:704–26. 10.1007/BF02804901

[4] Franks PJ . Recent advances in modelling of harmful algal blooms. In: Glibert PM, Berdalet E, Burford MA, Pitcher GC, Zhou M (eds), Global Ecology and Oceanography of Harmful Algal Blooms*.* Cham: Springer, 2018, 359–77. 10.1007/978-3-319-70069-4_19

[5] Glibert PM , Allen JI, Bouwman A et al. Modeling of HABs and eutrophication: status, advances, challenges. *J Mar Syst* 2010;83:262–75. 10.1016/j.jmarsys.2010.05.004

[6] Sellner KG , Doucette GJ, Kirkpatrick GJ. Harmful algal blooms: causes, impacts and detection. *J Ind Microbiol Biotechnol* 2003;30:383–406. 10.1007/s10295-003-0074-9 12898390

[7] Glibert PM , Burkholder JM. Causes of harmful algal blooms. In: Shumway SE, Burkholder JM, Morton SL (eds), Harmful Algal Blooms: A Compendium Desk Reference*.* Hoboken: John Wiley & Sons, 2018, 1–38. 10.1002/9781118994672.ch1

[8] Glibert PM . Harmful algae at the complex nexus of eutrophication and climate change. *Harmful Algae* 2020;91:101583. 10.1016/j.hal.2019.03.001 32057336

[9] Zhou J , Richlen ML, Sehein TR et al. Microbial community structure and associations during a marine dinoflagellate bloom. *Front Microbiol* 2018;9:1201. 10.3389/fmicb.2018.01201 29928265 PMC5998739

[10] Zhou J , Zhang BY, Yu K et al. Functional profiles of phycospheric microorganisms during a marine dinoflagellate bloom. *Water Res* 2020;173:115554. 10.1016/j.watres.2020.115554 32028248

[11] Doucette GJ . Interactions between bacteria and harmful algae: a review. *Nat Toxins* 1995;3:65–74. 10.1002/nt.2620030202 7613737

[12] Kuhlisch C , Shemi A, Barak-Gavish N et al. Algal blooms in the ocean: hot spots for chemically mediated microbial interactions. *Nat Rev Microbiol* 2024;22:138–54. 10.1038/s41579-023-00975-2 37833328

[13] Garcés E , Vila M, Reñé A et al. Natural bacterioplankton assemblage composition during blooms of *Alexandrium* spp.(Dinophyceae) in NW Mediterranean coastal waters. *Aquat Microb Ecol* 2007;46:55–70. 10.3354/ame046055

[14] Sun L , Gao P, Li Y et al. Microbial community coexisting with harmful alga *Karenia mikimotoi* and microbial control of algal bloom in laboratory. *J Oceanol Limnol* 2022;40:1027–38. 10.1007/s00343-021-1087-9

[15] Mayali X , Doucette GJ. Microbial community interactions and population dynamics of an algicidal bacterium active against *Karenia brevis* (Dinophyceae). *Harmful Algae* 2002;1:277–93. 10.1016/S1568-9883(02)00032-X

[16] Amin S , Hmelo L, Van Tol H et al. Interaction and signalling between a cosmopolitan phytoplankton and associated bacteria. *Nature* 2015;522:98–101. 10.1038/nature14488 26017307

[17] Bell W , Mitchell R. Chemotactic and growth responses of marine bacteria to algal extracellular products. *Biol Bull* 1972;143:265–77. 10.2307/1540052

[18] Seymour JR , Amin SA, Raina JB et al. Zooming in on the phycosphere: the ecological interface for phytoplankton–bacteria relationships. *Nat Microbiol* 2017;2:1–12. 10.1038/nmicrobiol.2017.65 28555622

[19] Tang YZ , Koch F, Gobler CJ. Most harmful algal bloom species are vitamin B_1_ and B_12_ auxotrophs. *Proc Natl Acad Sci USA* 2010;107:20756–61. 10.1073/pnas.1009566107 21068377 PMC2996436

[20] Lin S , Hu Z, Song X et al. Vitamin B_12_-auxotrophy in dinoflagellates caused by incomplete or absent cobalamin-independent methionine synthase genes (*metE*). *Fundam Res* 2022;2:727–37. 10.1016/j.fmre.2021.12.014 38933134 PMC11197592

[21] Croft MT , Lawrence AD, Raux-Deery E et al. Algae acquire vitamin B_12_ through a symbiotic relationship with bacteria. *Nature* 2005;438:90–3. 10.1038/nature04056 16267554

[22] Mayali X , Azam F. Algicidal bacteria in the sea and their impact on algal blooms^1^. *J Eukaryot Microbiol* 2004;51:139–44. 10.1111/j.1550-7408.2004.tb00538.x 15134248

[23] Coyne KJ , Wang Y, Johnson G. Algicidal bacteria: a review of current knowledge and applications to control harmful algal blooms. *Front Microbiol* 2022;13:871177. 10.3389/fmicb.2022.871177 35464927 PMC9022068

[24] Wang B , Yang X, Lu J et al. A marine bacterium producing protein with algicidal activity against *Alexandrium tamarense*. *Harmful Algae* 2012;13:83–8. 10.1016/j.hal.2011.10.006

[25] Lu X , Zhou B, Xu L et al. A marine algicidal *Thalassospira* and its active substance against the harmful algal bloom species *Karenia mikimotoi*. *Appl Microbiol Biot* 2016;100:5131–9. 10.1007/s00253-016-7352-8 26846742

[26] Anderson DM , Fensin E, Gobler CJ et al. Marine harmful algal blooms (HABs) in the United States: history, current status and future trends. *Harmful Algae* 2021;102:101975. 10.1016/j.hal.2021.101975 33875183 PMC8058451

[27] Tullis-Joyce P , Roy SS. Occurrence of *Karenia brevis* near Southwest Florida coast 1971 to 2017: a geospatial analysis. *J Coast Conserv* 2021;25:1–14. 10.1007/s11852-021-00844-1

[28] Flewelling LJ , Naar JP, Abbott JP et al. Red tides and marine mammal mortalities. *Nature* 2005;435:755–6. 10.1038/nature435755a 15944690 PMC2659475

[29] Glibert PM , Burkholder JM, Kana TM et al. Grazing by *Karenia brevis* on *Synechococcus* enhances its growth rate and may help to sustain blooms. *Aquat Microb Ecol* 2009;55:17–30. 10.3354/ame01279

[30] Van Dolah FM , Lidie KB, Monroe EA et al. The Florida red tide dinoflagellate *Karenia brevis*: new insights into cellular and molecular processes underlying bloom dynamics. *Harmful Algae* 2009;8:562–72. 10.1016/j.hal.2008.11.004

[31] Roth PB , Twiner MJ, Mikulski CM et al. Comparative analysis of two algicidal bacteria active against the red tide dinoflagellate *Karenia brevis*. *Harmful Algae* 2008;7:682–91. 10.1016/j.hal.2008.02.002

[32] Pokrzywinski KL , Place AR, Warner ME et al. Investigation of the algicidal exudate produced by *Shewanella* sp. IRI-160 and its effect on dinoflagellates. *Harmful Algae* 2012;19:23–9. 10.1016/j.hal.2012.05.002

[33] Bolyen E , Rideout JR, Dillon MR et al. Reproducible, interactive, scalable and extensible microbiome data science using QIIME 2. *Nat Biotechnol* 2019;37:852–7. 10.1038/s41587-019-0209-9 31341288 PMC7015180

[34] Brown CT , Howe A, Zhang Q et al. A reference-free algorithm for computational normalization of shotgun sequencing data. *ArXiv* 2012;**1203**:4802v2. 10.48550/arXiv.1203.4802

[35] Uritskiy GV , DiRuggiero J, Taylor J. MetaWRAP—a flexible pipeline for genome-resolved metagenomic data analysis. *Microbiome* 2018;6:158. 10.1186/s40168-018-0541-1 30219103 PMC6138922

[36] Wood DE , Lu J, Langmead B. Improved metagenomic analysis with Kraken 2. *Genome Biol* 2019;20:257. 10.1186/s13059-019-1891-0 31779668 PMC6883579

[37] Breitwieser FP , Salzberg SL. Pavian: interactive analysis of metagenomics data for microbiome studies and pathogen identification. *Bioinformatics* 2020;36:1303–4. 10.1093/bioinformatics/btz715 31553437 PMC8215911

[38] Li D , Liu C-M, Luo R et al. MEGAHIT: an ultra-fast single-node solution for large and complex metagenomics assembly via succinct de Bruijn graph. *Bioinformatics* 2015;31:1674–6. 10.1093/bioinformatics/btv033 25609793

[39] Chaumeil PA , Mussig AJ, Hugenholtz P et al. GTDB-Tk: a toolkit to classify genomes with the genome taxonomy database. *Bioinformatics* 2020;36:1925–7. 10.1093/bioinformatics/btz848 PMC7703759 31730192

[40] Pritchard L , Cock P, Esen Ö. Pyani v0.2.8: average nucleotide identity (ANI) and related measures for whole genome comparisons. *SIPBS* 2019. 10.5281/zenodo.594921 (2020, date last accessed).

[41] Bushnell B . BBMap: a fast, accurate, splice-aware aligner. OSTI.gov. https://www.osti.gov/biblio/1241166 (2014, date last accessed).

[42] Patro R , Duggal G, Love MI et al. Salmon provides fast and bias-aware quantification of transcript expression. *Nat Methods* 2017;14:417–9. 10.1038/nmeth.4197 28263959 PMC5600148

[43] Eren AM , Esen ÖC, Quince C et al. Anvi’o: an advanced analysis and visualization platform for ‘omics data. *PeerJ* 2015;3:e1319. 10.7717/peerj.1319 26500826 PMC4614810

[44] Blin K , Shaw S, Kloosterman AM et al. antiSMASH 6.0: improving cluster detection and comparison capabilities. *Nucleic Acids Res* 2021;49:W29–35. 10.1093/nar/gkab335 33978755 PMC8262755

[45] Paul C , Pohnert G. Interactions of the algicidal bacterium *Kordia algicida* with diatoms: regulated protease excretion for specific algal lysis. *PLoS One* 2011;6:e21032. 10.1371/journal.pone.0021032 21695044 PMC3117869

[46] Vernette C , Lecubin J, Sánchez P et al. The ocean gene atlas v2.0: online exploration of the biogeography and phylogeny of plankton genes. *Nucleic Acids Res* 2022;50:W516–26. 10.1093/nar/gkac420 35687095 PMC9252727

[47] Wickham H . ggplot2. *WIREs Comp Stats* 2011;3:180–5. 10.1002/wics.147

[48] Becker RA , Wilks AR, Brownrigg R et al. Maps: draw geographical maps R package version 3.2.0. https://CRAN.R-project.org/package=maps. 2017.;

[49] Sipler RE , McGuinness LR, Kirkpatrick GJ et al. Bacteriocidal effects of brevetoxin on natural microbial communities. *Harmful Algae* 2014;38:101–9. 10.1016/j.hal.2014.04.009

[50] Schofield O , Kerfoot J, Mahoney K et al. Vertical migration of the toxic dinoflagellate *Karenia brevis* and the impact on ocean optical properties. *J Geophys Res Oceans* 2006;111:C06009. 10.1029/2005JC003115

[51] McNichol J , Berube PM, Biller SJ et al. Evaluating and improving small subunit rRNA PCR primer coverage for bacteria, archaea, and eukaryotes using metagenomes from global ocean surveys. *Msystems* 2021;6:e0056521–128. 10.1128/msystems.00565-21 34060911 PMC8269242

[52] Rodriguez-R LM , Conrad RE, Viver T et al. An ANI gap within bacterial species that advances the definitions of intra-species units. *MBio* 2023;15:e0269623. 10.1128/mbio.02696-23 38085031 PMC10790751

[53] Geng H , Belas R. Molecular mechanisms underlying *Roseobacter*–phytoplankton symbioses. *Curr Opin Biotechnol* 2010;21:332–8. 10.1016/j.copbio.2010.03.013 20399092

[54] Buchan A , González JM, Moran MA. Overview of the marine *Roseobacter* lineage. *Appl Environ Microb* 2005;71:5665–77. 10.1128/AEM.71.10.5665-5677.2005 PMC1265941 16204474

[55] Cai Z , Liu Y, Chen Y et al. RpoN regulates virulence factors of *Pseudomonas aeruginosa* via modulating the PqsR quorum sensing regulator. *Int J Mol Sci* 2015;16:28311–9. 10.3390/ijms161226103 26633362 PMC4691050

[56] Huang L , Zhao L, Liu W et al. Dual RNA-Seq unveils *pseudomonas plecoglossicida htpG* gene functions during host-pathogen interactions with *Epinephelus coioides*. *Front Immunol* 2019;10:984. 10.3389/fimmu.2019.00984 31130962 PMC6509204

[57] Cho JC , Giovannoni SJ. *Croceibacter atlanticus* gen. Nov., sp. nov., a novel marine bacterium in the family Flavobacteriaceae. *Syst Appl Microbiol* 2003;26:76–83. 10.1078/072320203322337344 12747413

[58] Lovejoy C , Bowman JP, Hallegraeff GM. Algicidal effects of a novel marine *Pseudoalteromonas* isolate (class Proteobacteria, gamma subdivision) on harmful algal bloom species of the genera *Chattonella*, *Gymnodinium*, and *Heterosigma*. *Appl Environ Microb.* 1998;64:2806–13. 10.1128/AEM.64.8.2806-2813.1998 PMC106776 9687434

[59] Wang J , Yin X, Xu M et al. Isolation and characterization of a high-efficiency algicidal bacterium *Pseudoalteromonas* sp. LD-B6 against the harmful dinoflagellate *Noctiluca scintillans*. *Front Microbiol* 2022;13:1091561. 10.3389/fmicb.2022.1091561 36619989 PMC9814975

[60] Lee SO , Kato J, Takiguchi N et al. Involvement of an extracellular protease in algicidal activity of the marine bacterium *Pseudoalteromonas* sp. strain A28. *Appl Environ Microb* 2000;66:4334–9. 10.1128/AEM.66.10.4334-4339.2000 PMC92304 11010878

[61] Kohno D , Sakiyama Y, Lee SO et al. Cloning and characterization of a gene encoding algicidal serine protease from *Pseudoalteromonas* sp. strain A28. *J Environ Biotechnol* 2007;7:99–102.

[62] Bartolek Z , Creveld SG, Coesel S et al. Flavobacterial exudates disrupt cell cycle progression and metabolism of the diatom *Thalassiosira pseudonana*. *ISME J* 2022;16:2741–51. 10.1038/s41396-022-01313-9 36104452 PMC9666458

[63] Oh HM , Kang I, Ferriera S et al. Complete genome sequence of *Croceibacter atlanticus* HTCC2559T. *J Bacteriol* 2010;192:4796–7. 10.1128/jb.00733-10 20639333 PMC2937408

[64] Rose MM , Scheer D, Hou Y et al. The bacterium *pseudomonas protegens* antagonizes the microalga *Chlamydomonas reinhardtii* using a blend of toxins. *Environ Microbiol* 2021;23:5525–40. 10.1111/1462-2920.15700 34347373

[65] O’Brien J , McParland EL, Bramucci AR et al. Biogeographical and seasonal dynamics of the marine *Roseobacter* community and ecological links to DMSP-producing phytoplankton. *ISME Commun* 2022;2:16. 10.1038/s43705-022-00099-3 37938744 PMC9723663

[66] Amin SA , Green DH, Hart MC et al. Photolysis of iron–siderophore chelates promotes bacterial–algal mutualism. *Proc Natl Acad Sci USA* 2009;106:17071–6. 10.1073/pnas.0905512106 19805106 PMC2761308

[67] Ren C-Z , Gao H-M, Dai J et al. Taxonomic and bioactivity characterizations of *Mameliella alba* strain LZ-28 isolated from highly toxic marine dinoflagellate *Alexandrium catenella* LZT09. *Mar Drugs* 2022;20:321. 10.3390/md20050321 35621971 PMC9147911

[68] Danish-Daniel M , Ming GH, Noor MEM et al. Draft genome sequence of *Mameliella alba* strain UMTAT08 isolated from clonal culture of toxic dinoflagellate *Alexandrium tamiyavanichii*. *Genom Data* 2016;10:12–4. 10.1016/j.gdata.2016.08.015 27625991 PMC5011168

[69] Varasteh T , Moreira AP, Silva Lima AW et al. Genomic repertoire of *Mameliella alba* Ep20 associated with Symbiodinium from the endemic coral *Mussismilia braziliensis*. *Symbiosis* 2020;80:53–60. 10.1007/s13199-019-00655-x

[70] Stewart VN , Wahlquist H, Burkey R. Occurrence of vitamin B_12_ along the Gulf coast of Florida. *Red Tide Studies, Pinellas to Collier Counties, 1963–1966: A Symposium*, Ed. Hodges R, Florida Board of Conservation Marine Laboratory, Maritime Base, Bayboro Harbor, St. Petersburg, FL. 1967:79–84.

[71] Zhang Y , Zhou X, Tang W et al. Decoding the influence of bacterial community structure and algicidal bacteria in a *Karenia longicanalis* bloom event. *Front Mar Sci* 2023;10:1242319. 10.3389/fmars.2023.1242319

[72] Syhapanha KS , Russo DA, Deng Y et al. Transcriptomics-guided identification of an algicidal protease of the marine bacterium *Kordia algicida* OT-1. *MicrobiologyOpen* 2023;12:e1387. 10.1002/mbo3.1387 37877654 PMC10565126

[73] Van Tol HM , Amin SA, Armbrust E. Ubiquitous marine bacterium inhibits diatom cell division. *ISME J* 2017;11:31–42. 10.1038/ismej.2016.112 27623332 PMC5315476

[74] Lyu YH , Zhou YX, Li Y et al. Optimized culturing conditions for an algicidal bacterium *Pseudoalteromonas* sp. SP 48 on harmful algal blooms caused by *Alexandrium tamarense*. *MicrobiologyOpen* 2019;8:e00803. 10.1002/mbo3.803 30734515 PMC6692542

[75] Tsujiso H , Miyamoto K, Tanaka K et al. Cloning and sequence analysis of a protease-encoding gene from the marine bacterium *Alteromonas* sp. strain O-7. *Biosci Biotechnol Biochem* 1996;60:1284–8. 10.1271/bbb.60.1284 8987544

[76] Chen XL , Wang Y, Wang P et al. Proteases from the marine bacteria in the genus *Pseudoalteromonas*: diversity, characteristics, ecological roles, and application potentials. *Mar Life Sci Technol* 2020;2:309–23. 10.1007/s42995-020-00058-8

[77] Doucette GJ , McGovern ER, Babinchak JA. Algicidal bacteria active against *Gymnodinium breve* (Dinophyceae). I. Bacterial isolation and characterization of killing activity^1, 3^. *J Phycol* 1999;35:1447–54. 10.1046/j.1529-8817.1999.3561447.x

