# Supplementary material for: Friends and foes: symbiotic and algicidal bacterial influence on Karenia brevis blooms
Source: ISME Commun. 2024 Dec 18;5(1):ycae164. doi: 10.1093/ismeco/ycae164 (PMC11740886; doi:10.1093/ismeco/ycae164)
Supplement: SUPPLEMENTARY_INFORMATION_ycae164 [file supplementary_information_ycae164.docx]

**SUPPLEMENTARY INFORMATION**

**for**

**Friends and Foes: Symbiotic and Algicidal bacterial influence on *Karenia brevis* blooms**

Cong Fei^1^, Anne Booker^2,3^, Sarah Klass^4^, Nayani K. Vidyarathna^3^, So Hyun Ahn^5^, Amin R. Mohamed^1^, Muhammad Arshad^6^, Patricia M. Glibert^3^, Cynthia A. Heil^4^, Joaquín M. Martínez^2,3^, Shady A. Amin^1,6,7*^

^1^ Marine Microbiomics Lab, New York University Abu Dhabi, UAE

^2^ Bigelow Laboratory for Ocean Sciences, East Boothbay, ME, USA

^3^ University of Maryland Center for Environmental Science, Horn Point Laboratory, MD, USA

^4^ Mote Marine Laboratory & Aquarium, Sarasota, FL, USA

^5^ Biology Department, Woods Hole Oceanographic Institution, MA, USA

^6^ Bioinformatics Core, Center for Genomics and Systems Biology, New York University Abu Dhabi, UAE

^7^ Mubadala ACCESS Center, New York University Abu Dhabi, UAE

***Corresponding author**

**Address:** PO Box 129188, Saadiyat Island Campus, Abu Dhabi, United Arab Emirates Tel: +971 2 628 5743
**Email:** samin@nyu.edu

**Supplementary Method**

1. **Algal strains and growth conditions**

*K. brevis* strain CCMP2229 was isolated from the central Florida coast, near Manasota (27.0171°N -82.4763°W) and the strain CCMP2228 was acquired from Mote Marine Laboratory's New Pass Dock (27.3689°N -82.5825°W) near Sarasota. To explore the bacterial community consistently associated with *K. brevis* strains, we employed xenic cultures in our study. Cultures were cultivated in the L1-Si medium [1], using seawater from GoM offshore seawater, both of which were filtered through GF/F Whatman® filters, and diluted to a salinity of 30 with deionized water and autoclaved. The 0.2-μm-filtered nutrient stocks were added based on the L1-Si medium. Cultures were maintained in a temperature-controlled walking chamber at 20°C and exposed to cool white fluorescent lighting of approximately 100 µmol/m^2^/s, following a 12:12 L:D cycle.

1. **Challenge experiment design and sample preparation**

Collected bloom seawater was pre-filtered onto a 100 µm nylon mesh (Isopore), and then sequentially fractionated using a 1 µm filter for CH1 water samples and a 0.8 µm filter for CH2 water samples to remove phytoplanktons, followed by further filtration through 0.22 and 0.02 µm membrane filters (Isopore) to obtain a bacterial and viral sample, a viral sample, and particle-free seawater, respectively. Fifty-milliliter aliquots of each size-fractionated sample were then maintained at *in situ* temperatures to preserve environmental conditions until further analysis.

*K. brevis* cultures were maintained at the late stationary phase to resemble a natural bloom with a concentration of 1x10^4^ cells/ml. For each fraction, we conducted a 'challenge' to assess its effect on *K. brevis* cultures CCMP2229 (termed Challenge Experiment 1, CH1) and CCMP2228 (termed Challenge Experiment 2, CH2). For CH1, four experimental conditions were established: enriched bacterial and viral fraction (BF), enriched viral fraction (VF), seawater control, and a xenic *K. brevis* control group (refer to Fig. 1A), with each condition represented in duplicate, totaling 8 flasks. Similarly, for CH2, the same four conditions were prepared in triplicate, summing to 12 flasks. Except for the unamended control flasks, each received a 10% v/v addition of the appropriate size-fractionated seawater filtrate. Subsequent incubation in a temperature-controlled walking chamber at 20°C and exposure to cool white fluorescent lighting of approximately 100 µmol/m^2^/s, following a 12:12 L:D cycle to ensure consistent environmental conditions for all cultures.

For CH1, one ml aliquots were collected for enumerating bacteria cells on Days 1, 2, 5, and 7 post-inoculation from each flask, and on Days 9, 11, and 13 post-inoculation from the flasks inoculated with BF water, VF water, particle-free seawater, and non-inoculated controls. The samples were fixed with a 0.01% Pluronic® F-68 solution and glutaraldehyde (0.25% final concentration) (Sigma-Aldrich) and snap-frozen in liquid nitrogen within 30 from the collection before storing them at -80°C until further analysis by flow cytometry. Flow cytometric enumeration of bacteria was conducted following established protocols [2,3] using a FACScan flow cytometer (Beckton Dickinson, Franklin Lakes, NJ, USA), equipped with an air-cooled laser providing 50 mW at 488 nm with standard filter set-up and with the BD CellQuesttTM Pro software. Briefly, once thawed, a subsample was diluted (10 to 1000-fold) in 1× TE buffer (10:1 Tris-EDTA, pH 8.0) to ensure an adequate events per second rate during analysis and stained with SYBRTM Green I for about 30 min at room temperature. Data was acquired for one minute and bacteria were discriminated based on green fluorescence and side scatter signals. For BF group, on Day 9, the remaining cultures were filtered through a 0.22 µm filter, rapidly frozen in liquid nitrogen, and stored at -80°C for DNA extraction. Furthermore, a mixture of 1.6 ml from the BF flasks was combined with 0.4 ml of 75% v/v sterile glycerol (Sigma-Aldrich) for bacterial isolation and stored in cryovials for preservation. On Day 13, the same preservation process was conducted for the remaining treatments. For all other treatments, on Day 15, an aliquot of 1.6 ml from each flask was mixed with 0.4 ml glycerol for storage.

For CH2, sampling occurred on Days 1, 2, 7, 8, 10, 14, and 15, following the same fixation protocol as CH1 for flow cytometry. Cultures were filtered on Day 8 for the BF and on Day 15 for other conditions through a 0.22 µm filter, with the subsequent steps identical to those of CH1.

1. **DNA extraction and sequencing**

DNA extraction was carried out for CH1 and CH2 with *K. brevis* cultures and from the monthly environmental samples using a DNeasy PowerWater Kit® (Qiagen, 14900) according to the manufacturer’s instructions but skipping the heating step to reduce *Karenia* lysis. DNA concentrations were quantified using a Quibit 4 Fluorometer (Thermo Fisher Scientific, MA, USA) and a NanoDrop2000 Spectrophotometer (Thermo Fisher Scientific, MA, USA). The absence of *K. brevis* DNA in our samples was confirmed by the lack of amplification using primers specific for the *Karenia* *rbcL* gene (forward primer, GATGATGARAAYATTAACTC; reverse primer, ATTTGTCCCGCATTGATTCCT) [4].

In the conducted experiments, both 16S rRNA amplicon and shotgun metagenomic sequencing were employed. For the 16S rRNA amplicon, sequencing was executed on the Illumina NovaSeq 6000 platform using a paired-end 250bp strategy. The entire process, from library preparation to quality control, was conducted by Novogene (HK, China), yielding over 100k raw reads per sample. For metagenomic analysis, shotgun sequencing was performed on both CH and monthly seawater samples. The xGen ssDNA-LowInput DNA library prep kit was utilized for sample preparation. CH1 samples were sequenced using the NovaSeq 6000 platform at Novogene (HK, China), achieving 150 Gb and 1 billion reads each. Meanwhile, CH2 and monthly samples were processed at the University of Illinois at Urbana-Champaign Roy J. Carver Biotechnology Center, USA, yielded 20 Gb and 50 million reads each.

1. **16S rRNA gene amplicon data analysis**

The microbial community composition derived from 16S rRNA amplicon sequencing was analyzed using the Quantitative Insights into Microbial Ecology (QIIME2) pipeline [5]. DADA2 algorithm [6] was used for quality control and removal of chimeric sequences, yielding an amplicon sequence variants (ASVs) feature table (Table S3). Taxonomic classifications for ASVs were determined by aligning them against the SILVA version 138 reference database via VSEARCH [7]. Subsequent analyses, including Principal Coordinates Analysis (PCoA) and hierarchical clustering, were carried out with the "vegan" package in R [8], based on the Bray-Curtis dissimilarity matrix. Data visualization was done using the "ggplot2" package in R version 4.2.2 [9].

1. **Shotgun metagenomic data analysis**

To ensure adequate metagenomic microbial reads and to account for the potential abundance of *K. brevis* reads, we undertook deep sequencing for the CH1 samples. The vast dataset from CH1 was digitally normalized using Khmer software [10], which helped in mitigating sequencing errors, removing redundant data, and standardizing sample variations. Based on results from CH1, sequencing depth was optimized for subsequent samples. CH2 and monthly seawater samples, with lesser data volume, did not necessitate digital normalization. Subsequently, all sample datasets underwent quality control and read trimming, including human contamination and adapter removal, using the Read_QC module from the metaWRAP pipeline with default parameters [11]. Taxonomic identification based on clean reads was conducted at the reads level using Kraken 2 with its standard database [12]. The class and species abundance matrix were visualized with Pavian [13]. Community composition was depicted using barplot and principal coordinates analysis (PCoA) plots in R.

Co-assembly was performed for CH1 and CH2 experiments using the MEGAHIT v1.1.2 assembler with default setting [14]. Initial binning was done using the metaWRAP binning module (with the --maxbin2 --concoct --metabat2 options). These initial bins from individual assemblies were then refined and consolidated into final metagenome-assembled genomes (MAGs) set following the bin_refinement module and bin_reassembly module of metaWRAP specifying a minimum 80% completion and a maximum 10% contamination (-c 80 -x 10 options). The phylogenomic tree was constructed with Anvi’o v7 based on 71 default bacterial single-copy core genes across 181 MAGs [15]. The phylogenetic tree was visualized and modified using the Interactive Tree Of Life (iTOL v5) tool [16].

1. **MAGs quantification**

Quantification of the MAGs was achieved using two distinct methodologies: Salmon and BBsplit, both of which were applied for read mapping and quantification purposes. For the Salmon-based quantification, MAG bins were processed using the Quant_bins module within the metaWRAP toolkit, set to its default parameters [11]. This module first determined the contig read depth for each sample and then proceeded to calculate the weighted contig abundance. This calculation involved multiplying the depth of each contig by its length, followed by normalizing these values against the total contig abundance within each sample. For the BBsplit approach, reads from bloom samples were mapped against the MAGs from the CH experiments to ascertain their relative abundance [17]. Data visualization was accomplished via heatmaps constructed with the 'pheatmap' package [18] in R, which helped to illustrate the compositional differences across experimental conditions.

1. **Pangenome analysis and functional enrichment**

For the pangenomic analysis, seventeen MAGs and three selected genomes were processed using the pangenomic workflow in anvi’o v7 [15]. Initially, the MAGs were converted from FASTA format to Anvi'o databases using the command ‘anvi-gen-contigs-database’. Subsequently, these databases were annotated with functional information derived from NCBI’s Clusters of Orthologous Groups (COGs) and the KEGG database, utilizing the commands ‘anvi-run-ncbi-cogs’ and ‘anvi-run-kegg-kofams’. To facilitate comprehensive genomic analysis, a genome storage database was created using ‘anvi-gen-genomes-storage’, incorporating genomic sequences, amino acid sequences, and functional annotations. The pangenomic landscape, encompassing core and accessory genomes, was constructed by executing ‘anvi-pan-genome’ with parameters ‘--minbit 0.5’, ‘--mcl-inflation 1.5’, and ‘--min-occurrence 5’. Visualization of the resulting pangenome was achieved through ‘anvi-display-pan’. Cluster analysis was determined by the presence or absence of gene clusters within the genomes. Furthermore, 'anvi-compute-functional-enrichment' was used to define gene functions across groups, identifying unique genes per group. Enrichment scores were calculated using a binomial generalized linear model and Rao's test statistic, with functions showing a q-value below 0.05 considered 'enriched' [19]. Each group's unique genes were identified and labeled.

1. **Bacterial isolation and identification**

For bacterial isolation, 200 µl aliquots from 2 ml glycerol stocks were spread onto various agar media to cultivate a diverse array of bacteria. These media included marine agar [20], marine agar supplemented with gentamicin, kanamycin, and ampicillin (each at 20 µg/ml), and agar infused with *K. brevis* culture filtrates. This strategic selection of different agar types was aimed at maximizing bacterial diversity by catering to unique bacterial resistances and growth conditions. Following the inoculation, the agar plates were incubated at 26°C for a period of up to five days. Post-incubation, single bacterial colonies were individually isolated and subjected to overnight culture. Subsequently, these cultures were snap-frozen in liquid nitrogen and stored at -80°C for long-term preservation.

Fresh bacterial cultures were initiated by plating on marine agar and incubating in marine broth at 26°C with agitation at 180 rpm. For genomic DNA extraction, 3 ml of the bacterial cultures were centrifuged, and the resulting pellets were processed using the Qiagen DNA Blood & Tissue kit. The 16S rRNA gene was then amplified from the extracted DNA, employing the universal primers 27F and 1492R. Amplification products were sequenced using an ABI 3730 DNA Analyzer (Apical Scientific, Malaysia). The acquired 16S rRNA gene sequences were first subjected to quality trimming using Seqman (Version 12, DNASTAR, Inc., Madison, WI, U.S.). Subsequently, alignment was performed with BioEdit version 7.2 [21], followed by phylogenetic analysis in MEGA X [22]. This analysis utilized the maximum likelihood method, incorporating the General Time Reversible (GTR) model. The generated phylogenetic trees were then visualized and annotated using iTOL v5 [16].

1. **Genome assembly**

The initial quality control of the raw sequences involved the removal of adapters: Fastp v0.23.4 [23] was utilized for trimming Illumina short reads, and NanoStat [24] was used for processing PacBio long reads. This process began with the assembly of short-read data via SPAdes v3.14.0 [25]. Subsequently, the assembly was enhanced by incorporating the long-read data, employing Miniasm [26] and Racon [27] for rapid assembly and error correction. Further refinement was achieved using Pilon [28], which provided additional polishing to enhance base-level accuracy. Finally, the assembly graph was visualized and analyzed using Bandage v0.9.0 [29], ensuring an accurate and comprehensive representation of the final genome structure.

1. **Algicidal activity of protesae in filtrates**

Overnight cultures of *P. spongiae* CE15 (OD> 0.9) in Marine Broth (MB) medium were diluted 1:200 in L1-Si medium. After 24h incubation, the cultures were gently filtered through a 0.22 µm filter (Isopore). To inhibit protease activity, cell-free filtrates were treated with 0.1 mM irreversible serine-protease inhibitor phenylmethanesulphoyfluoride (PMFS; Sigma, Germany) and incubated in the dark at room tempurature for 1 hour. Protease activity was measured using the BODIPY TR-X casein (EnzChek Protease Assay Kit, Invitrogen) according to the manufacturer’s instructions. Fluorescence intensity was read using a BioTek Cytation 5 plate reader (Ex/Em = 590/645 nm). For the algicidal test, K. brevis CCMP2229 was inoculated into 3 mL of filtrates at a concentration of 1x10^3^ cells/mL in 24-well plates, with triplicate samples. Cell counts were measured by flow cytometry (CyFlow Space, SYSMEX) after 5 hours. Controls for the protease assay included untreated filtrates, L1-Si medium with MB, L1-Si medium, and L1-Si medium with 0.1 mM PMSF.

1. ***In situ* experiment**

*In situ* onboard experiments were carried out on the ECOHAB process cruise (PC0223), with seawater sampled at station TC16 (latitude 26.4763, longitude -82.2015) on February 9th 2023 using a General Oceanics rosette equipped with 12-liter Niskin bottles and CTD sensors. Six flasks, each with 200 ml of seawater, were set up for the experiment. *C. atlanticus* CE21, grown on MB agar, was resuspended to 1×10^6^ cells/ml and inoculated into the flasks in triplicate to evaluate the effects on *K. brevis*. Flasks were incubated on deck under near-ambient temperatures and 50% surface light irradiance. *K. brevis* cell counts were recorded at 0, 16, and 24 hours under a Zeiss Primo-Start Compound Microscope.

**Supplementary Information References**

1. Guillard R, Hargraves P. *Stichochrysis immobilis* is a diatom, not a chrysophyte. *Phycologia*. 1993;32(3):234-6.

2. Brussaard CP. Optimization of procedures for counting viruses by flow cytometry. *Appl Environ Microb*. 2004;70(3):1506-13.

3. Marie D, Brussaard CP, Thyrhaug R et al. Enumeration of marine viruses in culture and natural samples by flow cytometry. *Appl Environ Microb*. 1999;65(1):45-52.

4. Gray M, Wawrik B, Paul J, Casper E. Molecular detection and quantitation of the red tide dinoflagellate *Karenia brevis* in the marine environment. *Appl Environ Microb*. 2003;69(9):5726-30.

5. Bolyen E, Rideout JR, Dillon MR et al. Reproducible, interactive, scalable and extensible microbiome data science using QIIME 2. *Nat Biotechnol*. 2019;37(8):852-857.

6. Callahan BJ, McMurdie PJ, Rosen MJ et al. DADA2: High-resolution sample inference from Illumina amplicon data. *Nat Methods*. 2016;13(7):581-3.

7. Rognes T, Flouri T, Nichols B et al. VSEARCH: a versatile open source tool for metagenomics. *PeerJ*. 2016;4:e2584.

8. Oksanen J, Blanchet FG, Kindt R et al. Package ‘vegan’. *Community ecology package, version*. 2013;2(9):1-295.

9. Wickham H. ggplot2. *Wiley interdisciplinary reviews: computational statistics*. 2011;3(2):180-5.

10. Brown CT, Howe A, Zhang Q et al. A reference-free algorithm for computational normalization of shotgun sequencing data. *arXiv preprint arXiv:12034802*. 2012;

11. Uritskiy GV, DiRuggiero J, Taylor J. MetaWRAP—a flexible pipeline for genome-resolved metagenomic data analysis. *Microbiome*. 2018;6(1):1-13.

12. Wood DE, Lu J, Langmead B. Improved metagenomic analysis with Kraken 2. *Genome Biol*. 2019;20:1-13.

13. Breitwieser FP, Salzberg SL. Pavian: interactive analysis of metagenomics data for microbiome studies and pathogen identification. *Bioinformatics*. 2020;36(4):1303-4.

14. Li D, Liu C-M, Luo R et al. MEGAHIT: an ultra-fast single-node solution for large and complex metagenomics assembly via succinct de Bruijn graph. *Bioinformatics*. 2015;31(10):1674-6.

15. Eren AM, Esen ÖC, Quince C et al. Anvi’o: an advanced analysis and visualization platform for ‘omics data. *PeerJ*. 2015;3:e1319.

16. Letunic I, Bork P. Interactive Tree Of Life (iTOL) v5: an online tool for phylogenetic tree display and annotation. *Nucleic Acids Res*. 2021;49(W1):W293-6.

17. Bushnell B. BBMap: a fast, accurate, splice-aware aligner. 2014;

18. Kolde R. Pheatmap: pretty heatmaps. *R package version*. 2012;1(2):726.

19. Shaiber A, Willis AD, Delmont TO et al. Functional and genetic markers of niche partitioning among enigmatic members of the human oral microbiome. *Genome Biol*. 2020;21:1-35.

20. Amin S, Hmelo L, Van Tol H et al. Interaction and signalling between a cosmopolitan phytoplankton and associated bacteria. *Nature*. 2015;522(7554):98-101.

21. Hall T, Biosciences I, Carlsbad C. BioEdit: an important software for molecular biology. *GERF Bull Biosci*. 2011;2(1):60-1.

22. Kumar S, Stecher G, Li M et al. MEGA X: molecular evolutionary genetics analysis across computing platforms. *Mol Biol Evol*. 2018;35(6):1547.

23. Chen S, Zhou Y, Chen Y et al. fastp: an ultra-fast all-in-one FASTQ preprocessor. *Bioinformatics*. 2018;34(17):i884-90.

24. De Coster W, D’hert S, Schultz DT et al. NanoPack: visualizing and processing long-read sequencing data. *Bioinformatics*. 2018;34(15):2666-9.

25. Bankevich A, Nurk S, Antipov D et al. SPAdes: a new genome assembly algorithm and its applications to single-cell sequencing. *Journal of computational biology*. 2012;19(5):455-477.

26. Li H. Minimap and miniasm: fast mapping and de novo assembly for noisy long sequences. *Bioinformatics*. 2016;32(14):2103-10.

27. Vaser R, Sović I, Nagarajan N et al. Fast and accurate de novo genome assembly from long uncorrected reads. *Genome Res*. 2017;27(5):737-46.

28. Walker BJ, Abeel T, Shea T et al. Pilon: an integrated tool for comprehensive microbial variant detection and genome assembly improvement. *PloS one*. 2014;9(11):e112963.

29. Wick RR, Schultz MB, Zobel J et al. Bandage: interactive visualization of de novo genome assemblies. *Bioinformatics*. 2015;31(20):3350-2.

**Supplementary figures**

**Figure S1.** **Growth dynamics of *K. brevis* and its microbial consortium.** (A) Cell densities of *K. brevis* in CH2 for each condition. (B) Total bacterial cell densities in CH2 across each condition. Arrows indicate the time points selected for DNA extraction. Error bars represent the s.d. of triplicate cultures.


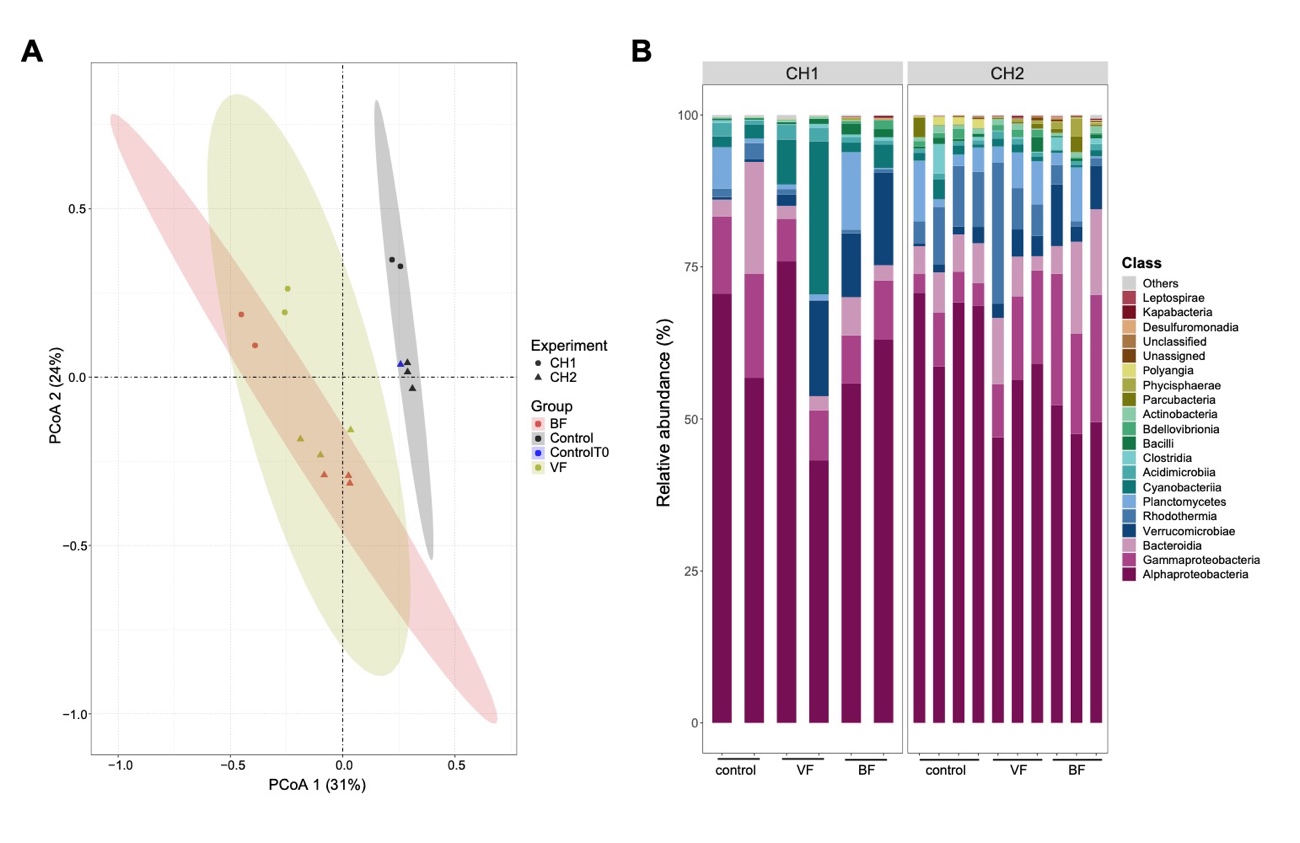
**Figure S2. Bacterial diversity and composition across CH1 and CH2.** (A) Principal coordinate analysis (PCoA) based on 16S rRNA ASVs for CH1 (circles) and CH2 (triangles) experiments. Dots are colored according to their respective groups, with 95% confidence ellipses depicted for each group (*P*=0.002). (B) Bar plot depicting the taxonomic composition of the top 20 bacterial classes in CH1 and CH2 based on 16S rRNA ASV data.


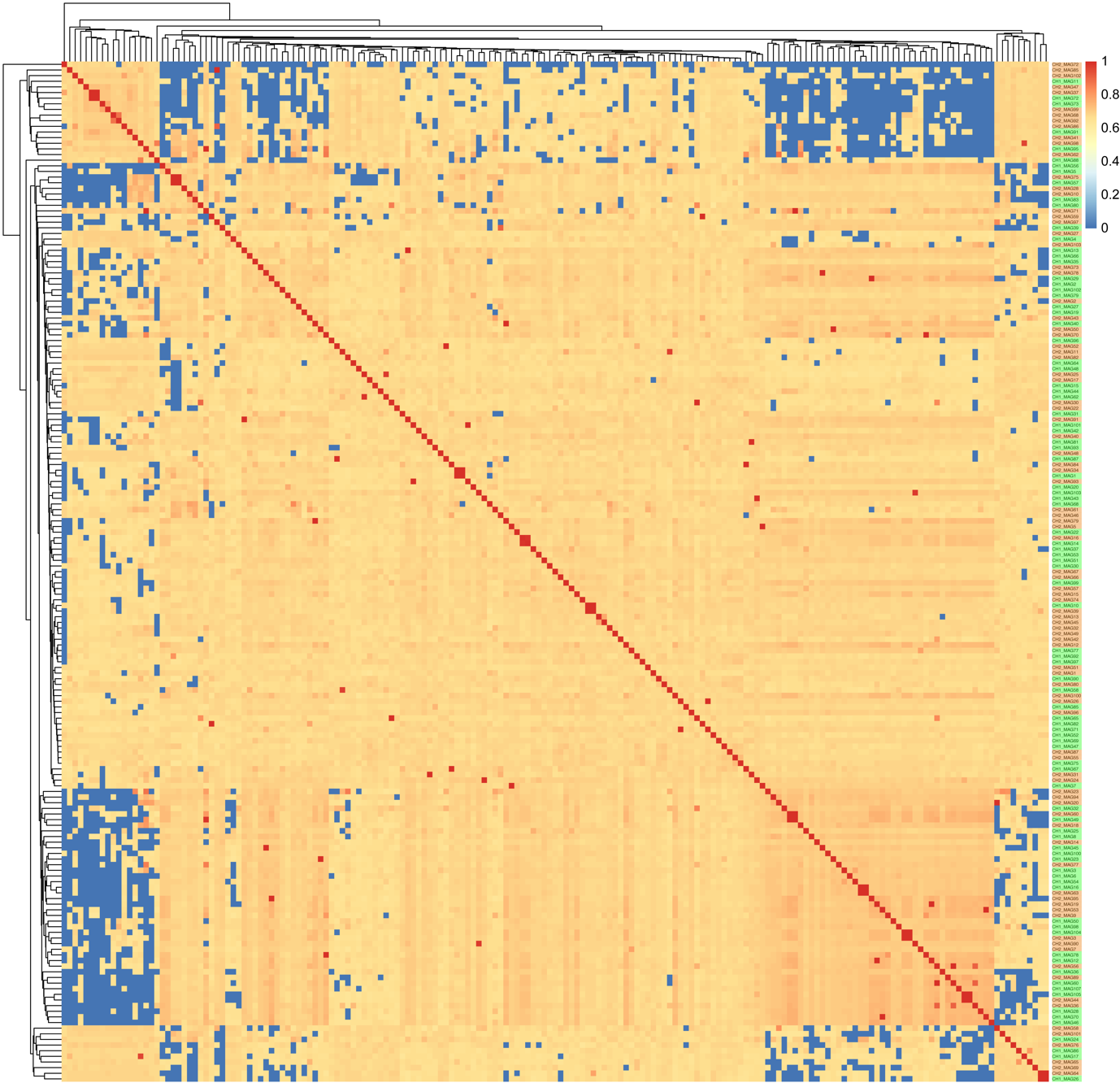
**Figure S3.** **Average nucleotide identity (ANI) values across 181 MAGs derived from CH1 and CH2.** The heatmap represents the results of BLASTn-based average nucleotide identity (ANIb), performed using PyANI. Green highlighted names indicate MAGs from CH1, while orange indicates MAGs from CH2.

**Figure S4. Effect of vitamins on *K. brevis* growth.** Vitamin-depleted *K. brevis* (*KB* - vitamins) cultures were supplemented with vitamins B_1_, B_7_, and B_12_, either combined or individually. The growth rate for *K. brevis* with all three vitamins (*KB* + vitamins) is shown. All error bars represent standard deviations from triplicate experiments.
